# Percussion, diuresis, and inversion therapy can facilitate lower renal pole stone passage after extracorporeal shock wave lithotripsy and retrograde intrarenal surgery? A systematic review and meta-analysis of randomized controlled trials

**DOI:** 10.1007/s00345-026-06656-y

**Published:** 2026-08-17

**Authors:** Luiz G. S. Gimenez, Giovanni S. Marchini, Fabio C. M. Torricelli, Alexandre Danilovic, Carlos A. Batagello, Fabio C. Vicentini, William C. Nahas, Eduardo Mazzucchi

**Affiliations:** Department of Urology, Hospital das Clinicas, University of Sao Paulo Medical School, Av. Dr. Eneas de Carvalho Aguiar, 255, 05403-000 Sao Paulo, SP Brazil

**Keywords:** Lithotripsy, Meta-analysis, Systematic review, Urolithiasis, Ureteroscopy

## Abstract

**Purpose:**

Patients with lower pole stones (LPS) present a significant challenge for urologists because of the increased likelihood of residual fragments and recurrence following extracorporeal shockwave lithotripsy (ESWL) and retrograde intrarenal surgery (RIRS). Although percussion, diuresis, and inversion (PDI) therapy has demonstrated potential for enhancing the stone-free rate (SFR) in these patients, its application remains a topic of debate. Consequently, our objective was to conduct a systematic review and meta-analysis of randomized controlled trials (RCTs) to evaluate SFR outcomes when comparing PDI therapy with observation in patients with LPS.

**Materials and methods:**

A systematic search was performed using PubMed, Embase, and Cochrane Library. We excluded conference abstracts, non-RCT trials, and studies that used the Lithecbole device. The main outcome was SFR, which was defined per study as no visible stone or residual fragments ≤ 2–4 mm. Risk of bias was assessed using the Cochrane RoB-2 tool and statistical analysis was performed using Review Manager 5.4.1 (Cochrane Collaboration).

**Results:**

Five RCTs comprising a total of 394 patients with LPS were included in this meta-analysis. Of these, 208 (52.7%) underwent PDI therapy; within the PDI group, 60 (28.8%) underwent RIRS and 148 (71.1%) underwent ESWL. The follow-up period ranged from 2 weeks to 3 months. Three studies reported the mean infundibular-pelvic angle and infundibular length; there was no significant difference between PDI and control (25.9 vs. 24.8 mm; *p* = 0.83) and (69.1 vs. 69.9 degrees; *p* = 0.20), respectively. The meta-analysis revealed a statistically significant difference in SFR (OR 3.81; 95% CI 2.40, 6.06; *p* < 0.00001; I²=0%; Fig. 2). Furthermore, there was no significant difference in the overall complication rate between groups (OR 0.36; 95% CI 0.04, 3.56; *p* = 0.38; I²=0%; Fig. 3). No study reported complications of Clavien-Dindo grade III or higher.

**Conclusions:**

In conclusion, PDI therapy after ESWL or RIRS was associated with a higher short-term SFR in patients with LPS compared with observation. These findings suggest a potential benefit of PDI as an adjunctive strategy for patients with LPS.

## Introduction

The prevalence of urinary lithiasis ranges from 13% in the North American population to 9% in the European population, with increasing rates attributed to population growth, dietary changes, and the rising prevalence of obesity [1]. Additionally, stone disease exhibits a recurrence rate of 10–50%, with a significantly higher risk of recurrence following the second episode [2]. Among renal stones, lower pole stones (LPS) account for approximately 35% of cases [3]. LPS pose a significant clinical challenge due to the higher likelihood of residual fragments and recurrence, primarily attributable to the gravity-dependent position of the lower calyx and anatomical constraints that hinder effective stone expulsion and promote recurrent stone formation [4, 5]. 

These challenges contribute to a lower SFR following treatments such as ESWL or retrograde intrarenal surgery [6]. The SFR is influenced by factors such as stone size and anatomical features of the renal collecting system, including infundibular width, length, and infundibulopelvic angle [5, 7, 8]. 

To address these anatomical limitations and improve stone clearance, adjuvant therapies, such as percussion, diuresis and inversion (PDI) therapy, have been proposed following ESWL or RIRS [9–13]. Although PDI therapy has shown promise for improving SFR in patients with LPS, its clinical utility remains controversial and understudied. Therefore, we conducted a systematic review and meta-analysis of RCTs to evaluate the efficacy of PDI therapy compared to observation in improving SFR among patients with LPS following ESWL or RIRS.

## Materials and methods

This systematic review and meta-analysis followed the guidelines provided by the Cochrane Collaboration and the Preferred Reporting Items for Systematic Reviews and Meta-Analysis (PRISMA) statement [14, 15]. 

### Eligibility criteria

Inclusion in this meta-analysis was restricted to studies that met the following eligibility criteria: [1] RCTs; [2] including patients with lower pole stones or fragments; [3] comparing PDI with observation after ESWL or RIRS; and [4] reports on SFR.

Studies were excluded if they were: [1] non-randomized; [2] lacked a control group; [3] used the Lithecbole device as percussion therapy, or [4] were other types of studies, such as case reports and reviews.

### Search strategy and data extraction

We systematically searched PubMed, Embase, and Cochrane Central Register of Controlled Trials from inception to April 2026, employing the following search strategy: (“calculus” OR urolithiasis OR ureteroscopy OR “RIRS” OR “furs” OR “lithotripsy”) AND (“percussion” OR “inversion” OR “PDI” OR “vibration” OR “vibrational” OR “EPVL” OR “postural” OR “lithecbole”). We also manually searched the references from all included studies, as well as previous systematic reviews and meta-analyses, for additional studies. Two authors (L.G.S.G. and G.S.M.) independently extracted data using predefined search criteria and quality assessment. Disagreements were resolved via consultation with another author (E.M.). The prospective meta-analysis protocol was registered on PROSPERO, under the protocol CRD42025649673, on Feb 08, 2025 and updated on May 01, 2026, to include any study published after the first registered date.

The initial search yielded 902 records. After removing duplicates and screening the titles and abstracts, 12 studies were assessed for eligibility based on the inclusion criteria. Of these, 4 were non-randomized trials and 3 used the Lithecbole device as percussion therapy. At the end, 5 RCTs were included in the analysis.

### Endpoints

Efficacy outcomes included SFR. Secondary outcome included overall complication rate.

### Quality assessment

Quality assessment of RCTs was performed using the Cochrane Collaboration tool for assessing risk of bias in randomized trials (Table 1), in which studies were scored as high, low, or some concerns of bias in five domains: the randomization process, deviations from intended interventions, missing outcome data, measurement of the outcome, and selection of the reported result.

**Table 1:** Risk of bias summary for randomized studies (RoB 2)

Study	Bias from randomization process	Bias due to deviations from intended interventions	Bias due to missing outcome data	Bias in measurement of the outcomes	Bias in selection of the reported result	Overall risk of bias
Chiong 2005	Some concerns	Low	Low	Low	Low	Some concerns
Pace 2001	Some concerns	Low	Low	Low	Low	Some concerns
Patel 2024	Low	Low	Low	Low	Low	Some concerns
Saleem 2015	Some concerns	Low	Low	High	Low	High
Sarioglu 2023	Some concerns	Low	Low	Low	Low	Some concerns

No trial reported allocation concealment, which raised four trials to “some concerns” in the randomization domain. Outcome-assessor blinding was reported in Chiong et al. and Patel et al., Saleem et al. blinded only the SWL-delivery physician, not the radiologist reading the stone-free threshold, and was rated high risk for outcome measurement; Sarioglu et al. lacked assessor blinding but was rated low risk given its binary CT-based outcome. Overall, one trial (Saleem et al.) was judged high risk and four (Chiong et al., Pace et al., Patel et al. and Sarioglu et al.) some concerns; none reached overall low risk. Two independent authors (L.G.S.G. and G.S.M.) completed the risk of bias assessment. The authors resolved any disagreements by reaching consensus after discussing the reasons for the discrepancies.

### Statistical analysis

Frequencies and percentages were used to report categorical variables; means and standard deviation (SD) were used to report continuous variables. Odds ratios (OR) and Mean Difference (MD) were used to evaluate categorical and continuous data, respectively, and 95% confidence interval (CI) and p-value were calculated. P-values of less than 0.05 were considered statistically significant. Cochran’s Q test and I^2^ statistics were used to assess heterogeneity, I^2^ > 25% was considered significant for heterogeneity. We used the DerSimonian and Laird random-effects model. Review Manager 5.4 (Cochrane Centre, The Cochrane Collaboration, Denmark) was used for the statistical analysis. Data reported as medians and interquartile ranges were converted to means and SD using the Wan and Luo method [16, 17]. Missing SDs were estimated from confidence intervals, p-values, or t-values, as recommended by the Cochrane Handbook [14]. 

## Results

### Study selection and characteristics

Five RCTs were included in the meta-analysis, comprising a total of 394 patients [9–13]. Of these, 208 (52.7%) underwent PDI and 186 (47.2%) underwent observation. Within the PDI group, 148 patients (71.1%) were treated with ESWL and 60 (28.8%) with RIRS. Similarly, in the observation group, 132 patients (70.9%) received ESWL and 54 (29%) received RIRS. Regarding percussion techniques within the PDI group, 173 patients (83.1%) were treated with hand delivery percussion, and 35 patients (16.8%) were treated with mechanical percussion using a portable mechanical device [10].

**Fig. 1:**
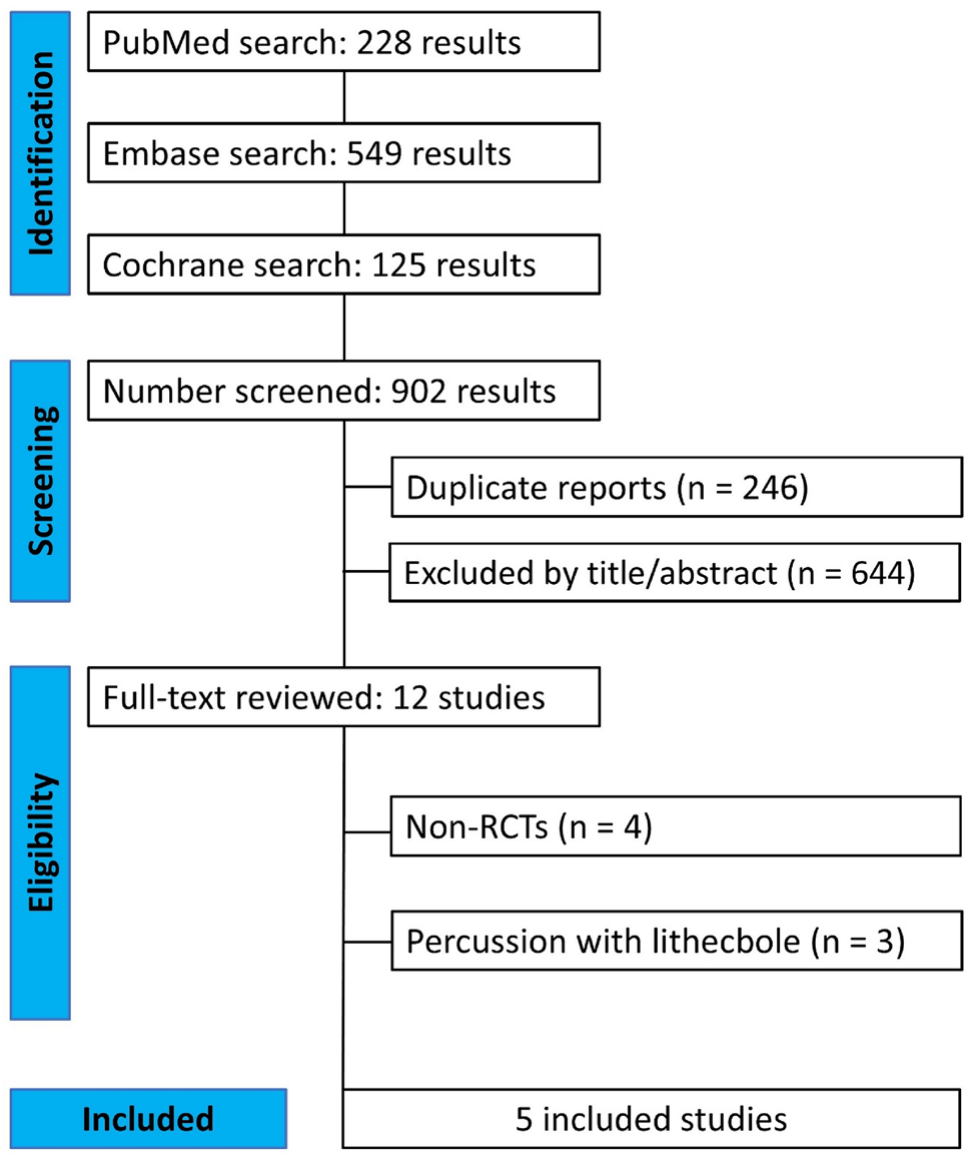
PRISMA flow diagram of study screening and selection

This distribution demonstrates a comparable profile between the two groups, with a predominance of patients treated with ESWL. The study selection process is detailed in Fig. 1 and the characteristics of the included studies are summarized in Table 2.

**Table 2:** Baseline characteristics of included studies

Study	Group	Patients, *n*	Surgery	Percussion device	SFR definition	Stone size	BMI, mean (SD)	Male, *n* (%)	IPA, degrees (SD)	IL, cm (SD)	IW, cm (SD)
Pace 2001	PDI	35	ESWL	Mechanical portable device	NVS at CT	61.7^a^	26.5 (4.4)	23 (65.7)	43.5 (18.4)	3.0 (0.7)	0.5 (0.3)
	Control	34				70.9^a^	25.2 (3.6)	29 (85.3)	40.2 (17.7)	3.1 (0.8)	0.4 (0.3)
Chiong 2005	PDI	59	ESWL	Hand	NVS at KUB	8 mm (4–20)^b^	25.8 (2.7)	50 (84.7)	99.4 (31.6)	2.2 (0.6)	0.4 (0.2–2.1)^b^
	Control	49				10 mm (4–20)^b^	25.1 (4.2)	30 (61.2)	107.5 (21.9)	2.1 (0.6)	0.5 (0.2–1.8)^b^
Saleem 2015	PDI	30	ESWL	Hand	RF < 2 mm at KUB	8–15^c^	NA	NA	NA	NA	NA
	Control	30				8–15^c^	NA	NA	NA	NA	NA
Sarioglu 2023	PDI	60	RIRS	Hand	NVS at CT	12.8 (2.6)^d^	28.7 (3.1)	35 (58.3)	52.5 (8.0)	2.4 (0.6)	0.50 (0.08)
	Control	54				12.5 (3.2)^d^	29.6 (4.1)	46 (85.1)	56.3 (9.3)	2.7 (0.5)	0.48 (0.18)
Patel 2024	PDI	24	ESWL	Hand	RF < 4 mm at KUB	5–10^c^	NA	NA	NA	NA	NA
	Control	19				5–10^c^	NA	NA	NA	NA	NA

BMI: body mass index; CT: computed tomography; ESWL: extracorporeal shock wave lithotripsy; IL: infundibular length; IPA: infundibulopelvic angle; IW: infundibular width; KUB: kidney, urinary, bladder x−ray; NA: not reported in the study; NVS: no visible stone; PDI: percussion, inversion and diuresis; RF: residual fragment; RIRS: retrograde intrarenal surgery; SD: standard deviation; SFR: stone−free rate

a: stone burden reported as mm². b: median (range). c: stone size range in mm, d: mean (SD)

Baseline characteristics were well-balanced between the PDI and observation groups, with no statistically significant differences observed in body mass index (BMI) (26.7 ± 3.4 vs. 27.2 ± 4.4 kg/m2; *p* = 0.27) or mean age (47.5 ± 9.9 vs. 44.6 ± 15.1 years; *p* = 0.051). Infundibular anatomy, as reported in three studies [9, 10, 13], also demonstrated comparability between groups, with no significant differences in mean infundibulopelvic angle (69.9 ± 32.5 vs. 69.1 ± 33.4 degrees; *p* = 0.83) or infundibular length (25.99 ± 7.38 vs. 24.89 ± 7.34 mm; *p* = 0.2). All studies included patients with LPS smaller than 2 cm. Details on differences in PDI techniques, such as inversion angle, time from lithotripsy to PDI, duration of PDI sessions, and follow-up, are presented in Table 3.

**Table 3:** Percussion, diuresis and inversion therapy (PDI) details

Study	Percussion	Surgery	First Session	Inversion (degree)	Water Ingestion (mL)	Diuretic	Duration (min)	Frequency / Duration	Follow up
Pace 2001	Mechanical percurssor	ESWL	> 3 M	60	NA	Furosemide 20 mg	10	1x / 4 W	1 M
Chiong 2005	Hand	ESWL	1–2 W	45	500	No	10	2 -4x / 1–2 W	3 M
Saleem 2015	Hand	ESWL	Immediate	15–30	500	Furosemide 20 mg	10	Once	2 W
Sarioglu 2023	Hand	RIRS	Immediate	30–45	500	No	20	Once	1 M
Patel 2024	Hand	ESWL	2 D	30–45	300	No	10	3x / 12 W	3 M

D: days; ESWL: Extracorporeal shockwave lithotripsy; M: month; RIRS: Retrograde intrarenal surgery; PDI: Percussion, inversion and diuresis;

NA: not available; W: week

### Meta-analysis results

#### Stone-free rate

Stone-free status was defined as residual fragments smaller than 4 mm. (Table 3) Evaluation methods for SFR varied across studies, encompassing KUB X-rays and CT scans. The SFR assessment timing ranged from 2 weeks to 3 months post-procedure.

**Fig. 2:**
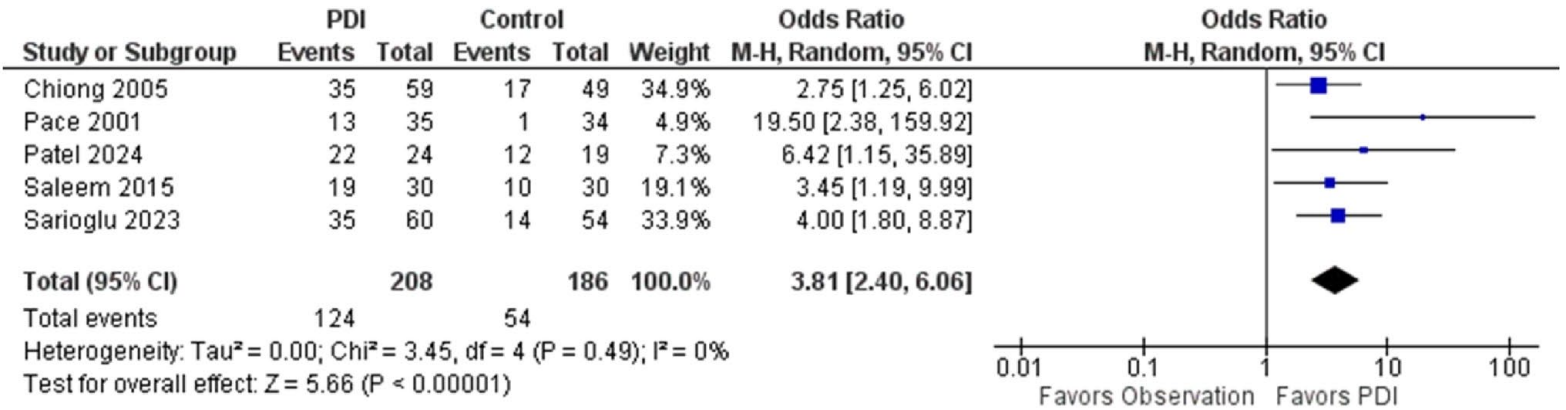
Stone free rate was significantly different between groups

The pooled SFR was 59.6% and 29% in the PDI and observation groups, respectively. The meta-analysis revealed a statistically significant difference in the SFR between the two groups, favoring PDI (OR 3.81; 95% CI 2.40, 6.06; *p* < 0.00001; I²=0%; Fig. 2).

#### Complication rate

**Fig. 3:**
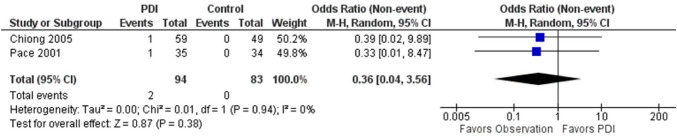
Complications was not significantly different between groups

The overall complication rate was similar between the PDI and observation groups (OR 0.36; 95% CI 0.04, 3.56; *p* = 0.38; I²=0%; Fig. 3). In the PDI group, the reported complications were limited to one patient experiencing pain during percussion therapy due to a minor kidney hematoma following ESWL [9] and one patient with a fragment migrating to the ureter, requiring analgesia and subsequent ESWL [10]. 

No Clavien-Dindo grade III or IV complications were observed in either group [18]. 

#### Subgroup analysis

The meta-analysis combines trials that differ across several methodological axes: lithotripsy methods (ESWL vs. RIRS), imaging modality for SFR assessment (KUB XR vs. CT), and stone free definition (no visible stone vs. residual-fragment threshold of < 2–4 mm), percussion technique (hand vs. mechanical device).

**Fig. 4:**
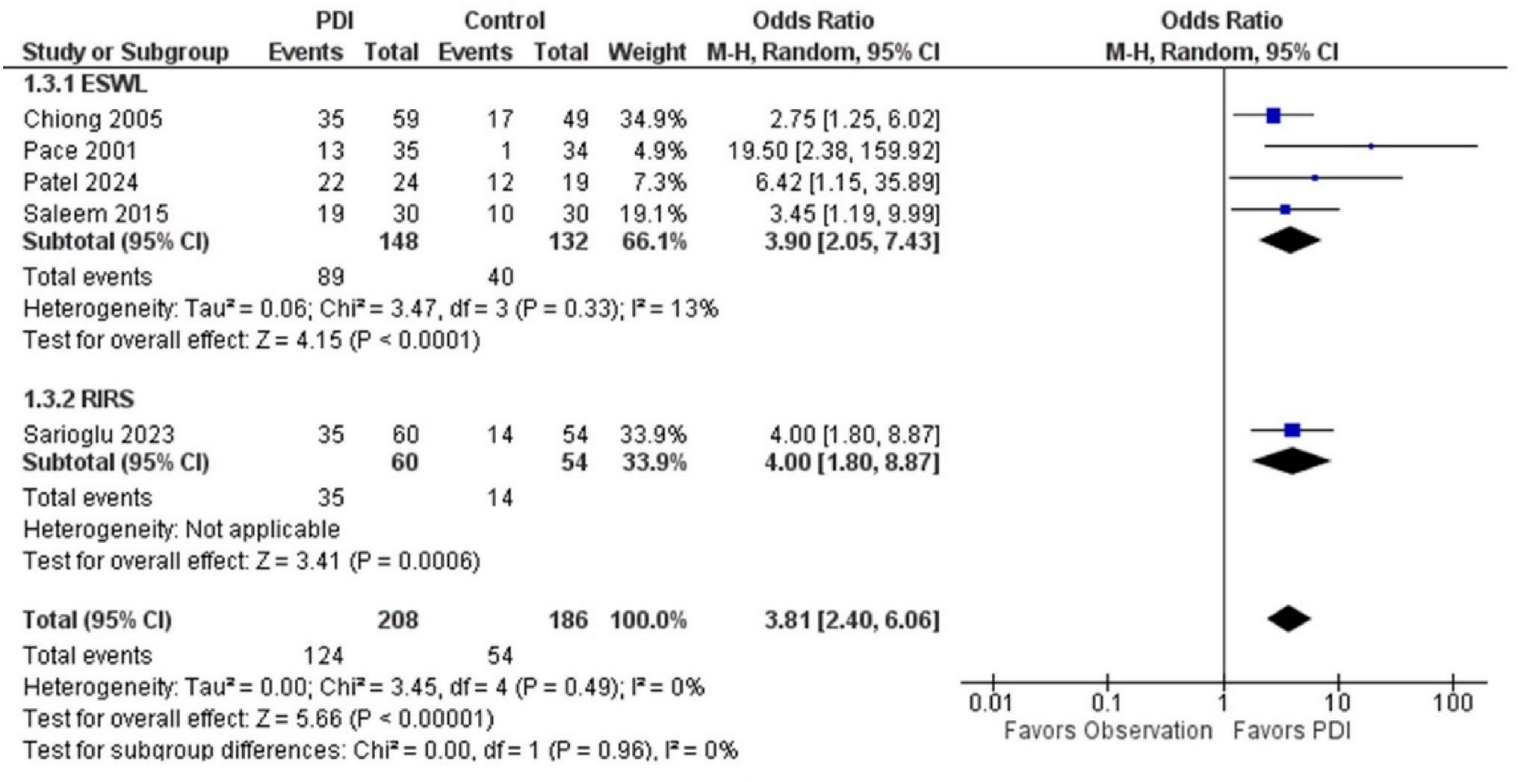
Subanalysis of lithotripsy method, stone free rate was significantly different between groups

**Fig. 5:**
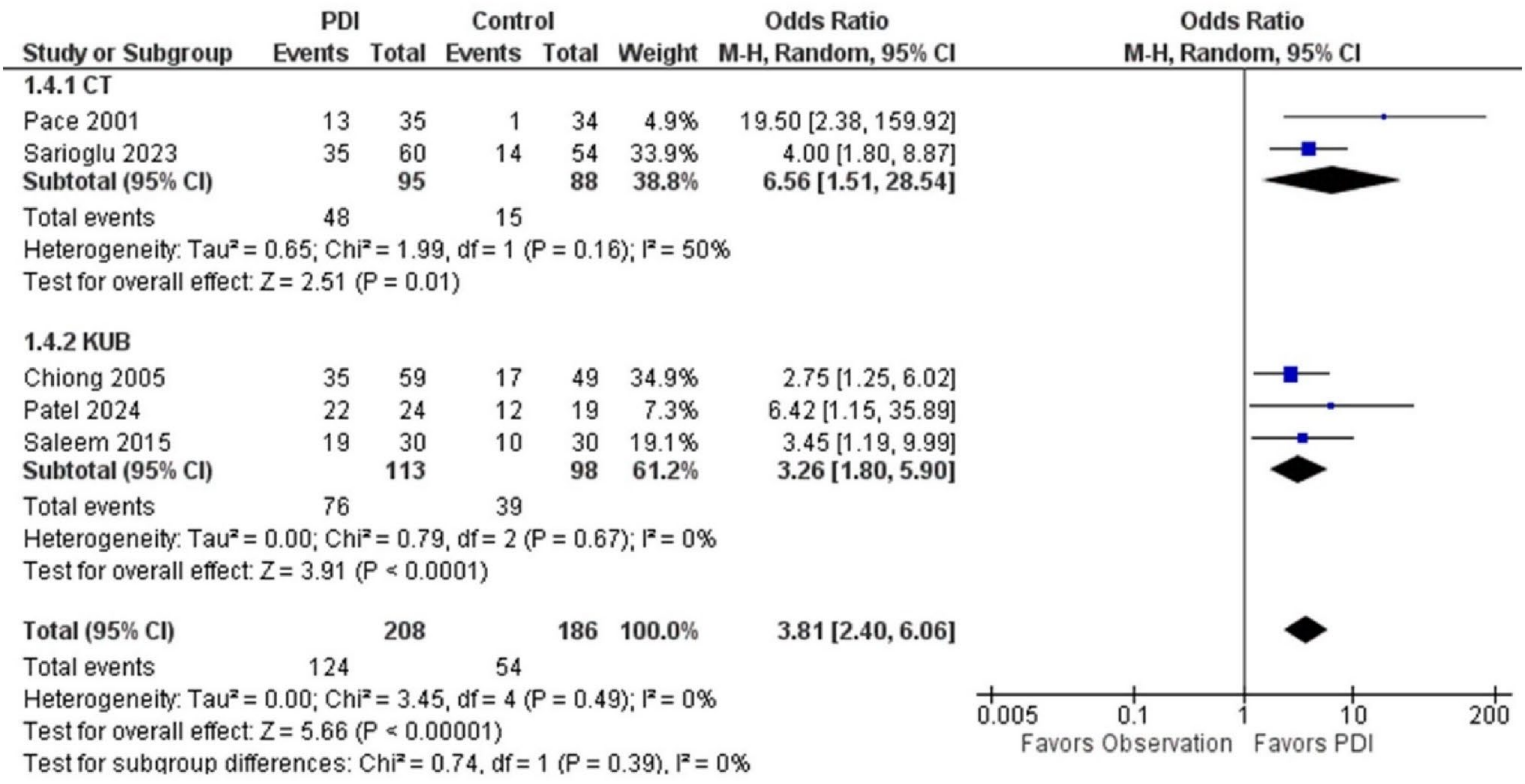
Subanalysis of imaging modality for stone free rate assessment

**Fig. 6:**
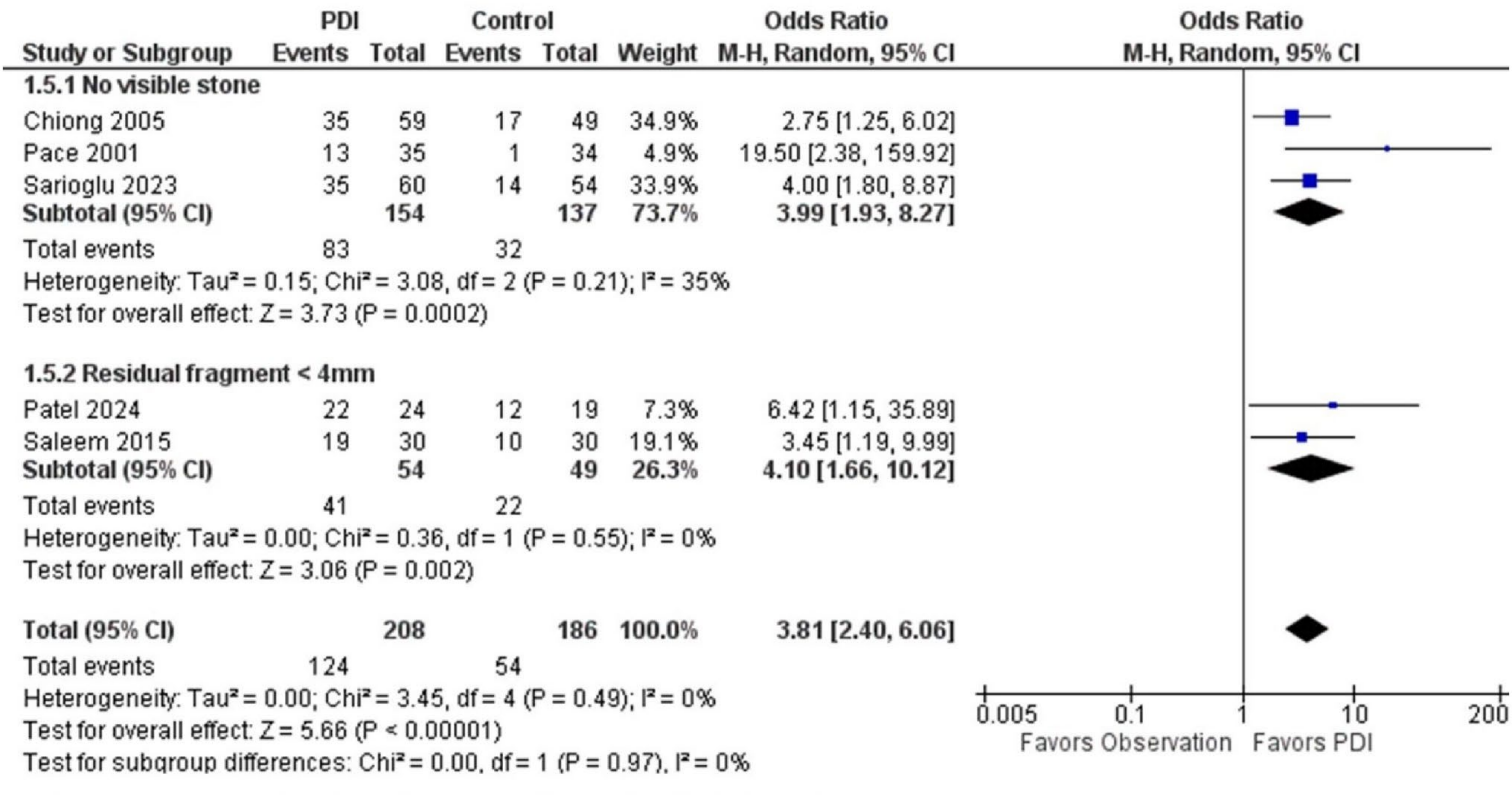
Subanalysis of stone free definition for stone free rate assessment

**Fig. 7:**
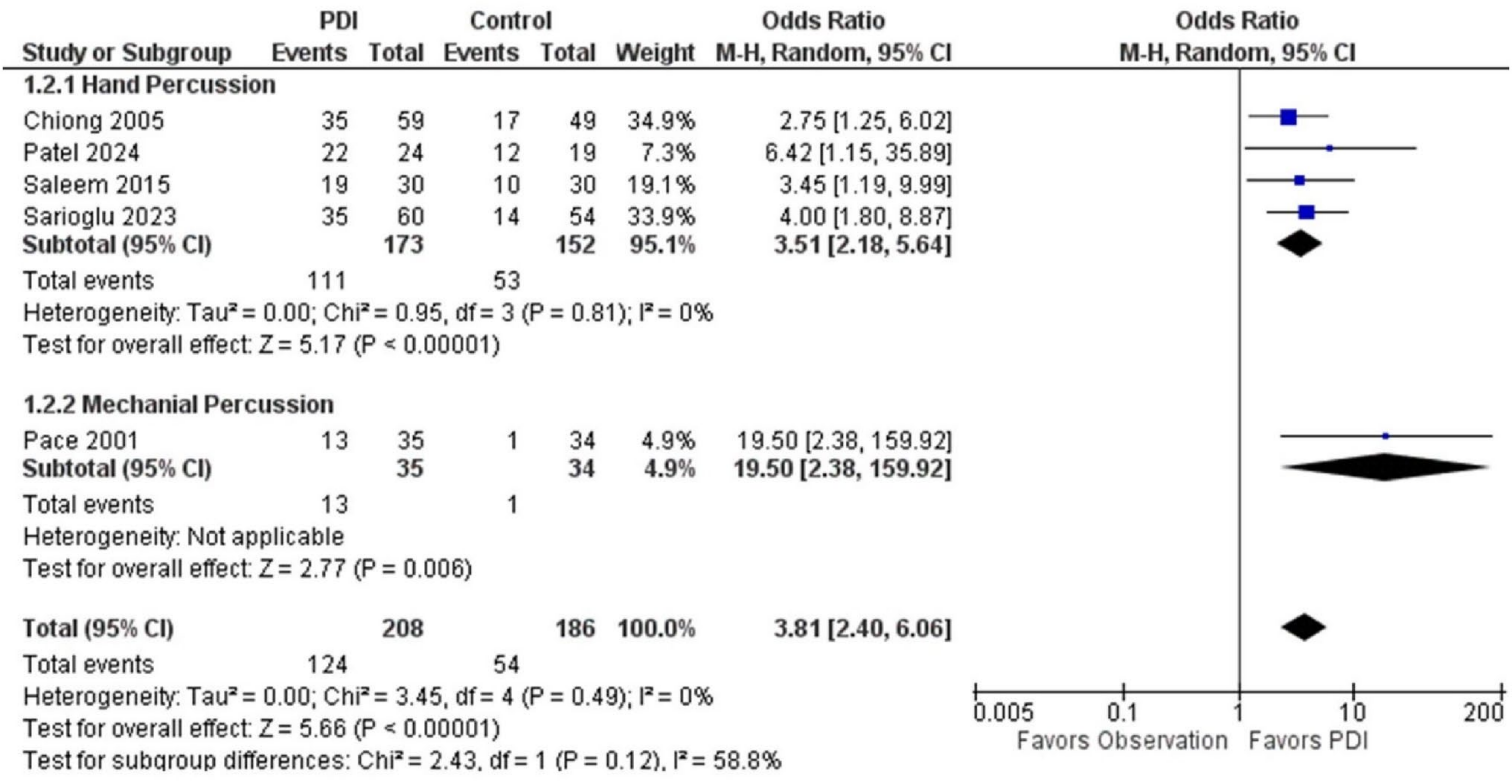
Subanalysis of percussion technique for stone free rate assessment

To assess whether this heterogeneity materially affected the pooled estimate, a subgroup analysis was performed for each factor: different lithotripsy methods (ESWL: OR 3.90; *p* < 0.0001 vs. RIRS: OR 4.00; *p* = 0.0006; Fig. 4), imaging modality for stone free assessment (KUB: OR 3.26; *p* < 0.0001 vs. CT: OR 6.56; *p* = 0.01; Fig. 5), stone free definition (NVS: OR 3.99; *p* = 0.0002 vs. RF: OR 4.10; *p* = 0.002; Fig. 6) and percussion technique (Hand: OR 3.51; *p* < 0.00001 vs. mechanical: OR 19.50; *p* = 0.006; Fig. 7) on the SFR.

As shown, SFRs were consistently higher in all PDI subgroups compared with observation. There were no significant differences between the subgroups, and heterogeneity was low.

## Discussion

We found a significantly elevated pooled SFR of 59.6% in the PDI group compared with 29% in the observation group (OR 3.81; *p* < 0.00001). This observation suggests a clinically meaningful benefit of PDI as an adjunctive therapy for promoting stone clearance following surgical intervention for LPS.

However, the pooled control-arm SFR of 29.0% reflects substantial between-study variability (range 2.9%–63.2%) rather than a single homogeneous rate. This variability is attributable to differences in SFR definition and imaging sensitivity: Pace et al., which used the strictest definition (no visible fragment on CT), reported a control-arm SFR of only 2.9% (1/34), whereas Patel et al., which used a more permissive residual-fragment threshold (< 4 mm on KUB), reported a control-arm SFR of 63.2% (12/19). These findings underscore that the low pooled control-arm SFR is a function of definitional and imaging heterogeneity across the included LPS-specific trials, rather than reflecting expected ESWL or RIRS stone-free rates in an unselected stone population.

Our findings are consistent with the broader literature on physical adjunctive therapy for lower pole stones. Peng et al. performed a meta-analysis of 8 studies (1,065 patients, including non-randomized studies) comparing physical therapy maneuvers with non-intervention and reported a similarly significant benefit in stone clearance (OR 3.38; 95% CI 2.45–4.66; *p* < 0.0001). The concordance between our RCT-restricted estimate and this broader analysis strengthens confidence in the direction and consistency of the PDI effect [19]. 

These findings are particularly notable given the challenges associated with achieving complete stone clearance in the anatomically complex lower pole region, where gravitational forces and infundibular geometry can impede fragment expulsion, a challenge highlighted by Sampaio and Aragao and further quantified in studies by Sarioglu et al. and Torricelli et al. [5, 7, 13]

The proposed mechanism of action for PDI therapy likely involves a synergistic interplay of biomechanical forces. Percussion may induce mechanical dislodgement of adherent calculi from renal calyces, as suggested by Honey et al. [20], while augmented fluid dynamics within the collecting system, secondary to induced diuresis, may facilitate the mobilization of stone fragments, a principle explored by Saleem et al. [12] Furthermore, gravitational forces, enhanced by patient inversion, may promote the expulsion of calculi from the dependent lower pole region, an approach initially investigated by Brownlee et al. [21]

Stone size is a recognized determinant of treatment success for lower pole stones and represents a potential confounder in this analysis. Stone-size data were reported in all included studies, but the units and format of reporting (stone burden in mm², median or mean values, or simple ranges) differed across studies precluding a formal meta-regression of stone size across the pooled cohort. Sarioglu et al. performed a multivariable analysis showing that the absence of PDI remained an independent predictor of treatment failure (OR 9.455; 95% CI 2.426–10.853; *p* = 0.001) after adjustment for stone size and infundibular length. This finding mitigates, though does not eliminate, the possibility of confounding by stone size.

Importantly, the meta-analysis did not reveal a statistically significant difference in complication rates between the PDI and observation groups. The observed complications in the PDI group were limited to Clavien-Dindo grade I or II with no grade III or IV complications. The pooled estimate of overall complication rate was derived from the two trials reporting at least one complication event in either arm (Chiong et al. and Pace et al.). Patel et al. reported complications only for the trial’s overall randomized population across all stone locations, not specifically for the lower-pole-stone subgroup included in this meta-analysis. Saleem et al. and Sarioglu et al. did not report on complications or adverse effects. Given the relatively small cumulative sample size and the selective reporting of adverse events, these results should be interpreted with caution.

From a practical standpoint, the PDI protocols evaluated in the included trials used low-cost, widely available components — manual or mechanical percussion, oral hydration with or without a diuretic, and postural inversion for 10–20 min — administered once to three times over a period of 1–12 weeks (Table 3). This suggests PDI is broadly feasible to implement in routine outpatient care without specialized equipment. However, none of the included trials performed a subgroup or interaction analysis to identify which patients benefit most from PDI; consequently, patient-selection criteria cannot be derived from the present evidence and represent an important area for future research. As with any postural intervention, standard precautions for head-down positioning should be observed in clinical practice.

## Limitations

The findings of this meta-analysis should be interpreted considering its limitations. The included studies used different imaging methods to establish SFR and employed diverse PDI protocols, with variations in the inversion angle, time from lithotripsy to PDI initiation, duration of PDI sessions and exhibited heterogeneity in the definition of stone-free status.

In addition, all five included trials reported stone size, but the units and format of reporting (stone burden in mm², median or mean values, or simple ranges) differed substantially across studies, precluding a formal meta-regression of stone size across the pooled cohort.

The mechanical-percussion subgroup analysis is based on a single trial with a small sample size (*n* = 69) and a wide confidence interval and should be interpreted with caution rather than as evidence of a distinct treatment effect for mechanical versus hand percussion. Complication reporting was inconsistent across trials; this heterogeneity introduces a risk of reporting bias, and the absence of a statistically significant difference in complications should be regarded as a preliminary, not definitive, safety signal.

Follow-up across the included trials was short, ranging from 2 weeks to 3 months, and none of the five RCTs reported stone recurrence as an outcome. Our findings are therefore restricted to short-term stone clearance and cannot be extrapolated to long-term, recurrence-free outcomes.

## Conclusion

This meta-analysis suggests that PDI therapy, when implemented as an adjunct to ESWL or RIRS, may improve short-term SFR in patients with LPS compared with observation, without a concomitant increase in overall complication rates. Given the limited number and size of available trials, this should be regarded as a potential rather than definitive benefit.

Future research should focus on standardizing PDI protocols, identifying optimal patient populations, and assessing the long-term impact of PDI therapy on stone recurrence rates and patient-reported outcomes.

## Data Availability

No datasets were generated or analysed during the current study.

## Abbreviations

BMI: Body mass index
ESWL: Extracorporeal shockwave lithotripsy
FURS: Flexible ureterorenoscopy
KUB: Kidney, urinary, bladder X ray
LPS: Lower pole stone
PDI: Percussion, diuresis and inversion
PRISMA: Preferred Reporting Items for Systematic Reviews and Meta–Analysis
RCT: Randomized controlled trial (s)
RIRS: Retrograde intrarenal surgery
OR: Odds ratio
SD: Standard deviation
SFR: Stone free rate
